# Development of Alcohol Use Disorder as a Function of Age, Severity, and Comorbidity with Externalizing and Internalizing Disorders in a Young Adult Cohort

**DOI:** 10.20900/jpbs.20190016

**Published:** 2019-10-25

**Authors:** John I. Nurnberger, Ziyi Yang, Yong Zang, Laura Acion, Laura Bierut, Kathleen Bucholz, Grace Chan, Danielle M. Dick, Howard J. Edenberg, John Kramer, Samuel Kuperman, John P. Rice, Marc Schuckit

**Affiliations:** 1 Departments of Psychiatry and Medical and Molecular Genetics and Stark Neurosciences Research Institute, Indiana University School of Medicine, Indianapolis, IN 46202, USA; 2 Department of Biostatistics, Indiana University School of Medicine, Indianapolis, IN 46202, USA; 3 Instituto de Calculo, Universidad de Buenos Aires-CONICET, Buenos Aires, C1428EGA, Argentina; 4 Department of Psychiatry, Washington University in St. Louis School of Medicine, St. Louis, MO 63110, USA; 5 Department of Psychiatry, University of Connecticut School of Medicine, Farmington, CT 06032, USA; 6 Department of Psychology, Virginia Commonwealth University, Richmond, VA 23284, USA; 7 Departments of Biochemistry and Molecular Biology and Medical and Molecular Genetics, Indiana University School of Medicine, Indianapolis, IN 46202, USA; 8 Department of Psychiatry, University of Iowa Carver College of Medicine, Iowa City, IA 52242, USA; 9 Department of Psychiatry, University of California San Diego School of Medicine, La Jolla, CA 92093, USA

**Keywords:** alcohol use disorders, high risk studies, family studies, externalizing disorders, internalizing disorders

## Abstract

**Background:**

As part of the ongoing Collaborative Study of the Genetics of Alcoholism, we performed a longitudinal study of a high risk cohort of adolescents/young adults from families with a proband with an alcohol use disorder, along with a comparison group of age-matched controls. The intent was to compare the development of alcohol problems in subjects at risk with and without comorbid externalizing and internalizing psychiatric disorders.

**Methods:**

Subjects (*N* = 3286) were assessed with a structured psychiatric interview at 2 year intervals over 10 years (2004–2017). The age range at baseline was 12–21.

**Results:**

Subjects with externalizing disorders (with or without accompanying internalizing disorders) were at increased risk for the onset of an alcohol use disorder during the observation period. Subjects with internalizing disorders were at greater risk than those without comorbid disorders for onset of a moderate or severe alcohol use disorder. The statistical effect of comorbid disorders was greater in subjects with more severe alcohol use disorders. The developmental trajectory of drinking milestones and alcohol use disorders was also accelerated in those with more severe disorders.

**Conclusions:**

These results may be useful for counseling of subjects at risk who present for clinical care, especially those subjects manifesting externalizing and internalizing disorders in the context of a positive family history of an alcohol use disorder. We confirm and extend findings that drinking problems in subjects at greatest risk will begin in early adolescence.

## INTRODUCTION

Alcohol problems typically develop in late adolescence and early adulthood, though they can manifest at any time during adult life. Early age at first drink has been shown in many analyses to be a powerful predictor of an alcohol use disorder (AUD) (see review in [1] Deutsch *et al.,* 2013). Family history of alcohol dependence is known to increase risk by at least two fold [2](Nurnberger *et al.,* 2004). Males are more likely than females to develop alcohol use disorders ([3] Hasin *et al.,* 2007; [4] Delker *et al.,* 2016; [5] Vasilenko *et al.,* 2017), and this is true within families of alcohol-dependent probands as well as the general population ([2] Nurnberger *et al.,* 2004). Recent data have shown that, in the US, African- Americans (AA) are less likely to develop an AUD than European- Americans (EA) ([6] Kessler *et al.,* 1994; [7] Smith *et al.,* 2006; [8] Huang *et al.,* 2006; [9] Grant *et al.,* 2015) though analysis over different age groups suggests that a different developmental course may characterize AUDs in African-Americans, with relatively later onset of disorders in comparison to EA groups ([10] Grant *et al.,* 2012; [5] Vasilenko *et al.,* 2017; [11] Liu and Mulia, 2018). It must be borne in mind that these rates are a moving target and there is evidence for relative increases of AUD in women and AA subjects compared to EA males over recent years ([12] Grant *et al*., 2017).

There is also a known risk relationship between other psychiatric disorders and alcohol use disorders. Persons with a mood disorder (especially bipolar disorder) have an increased lifetime risk for an alcohol use disorder, as compared with persons without mood disorders ([13] Glantz *et al.,* 2009). The increased risk for a substance use disorder (alcohol or drugs) following onset of a mood disorder is perhaps most precisely demonstrated by [14] Plana-Ripoll *et al.* 2019, using a study of the Danish population that showed a cumulative risk of 20% for men and 10% for women for an SUD during the fifteen years following the onset of a mood disorder. This represents a hazard ratio of ~5 for a disorder severe enough to come to clinical attention. Adolescents with a mood disorder are at increased risk for onset of alcohol problems ([15] Kessler *et al.,* 2012; [16] Boschloo *et al.,* 2013) and *vice versa* ([17] Kandel *et al.,* 1999). Mood disorder may be associated with the course of alcohol problems as well as onset ([18] Crum *et al.,* 2018). Scores on an internalizing scale were correlated with risk for alcohol and other drug use disorders in a prior analysis of the Collaborative Study on the Genetics of Alcoholism (COGA) subjects ([19] Acion *et al.,* 2019).

There is an extensive literature supporting the relationship of externalizing disorders to subsequent development of AUDs and this has formed the basis of certain typologies of AUD, including Types 1 and 2 ([20] Cloninger, 1987) and Types A and B ([21] Babor *et al.,* 1992). Type 2 subjects are characterized by high novelty seeking, low harm avoidance, and low reward dependence ([20] Cloninger, 1987). They are more likely to be diagnosed with antisocial personality disorder and less likely to be able to abstain from alcohol. Type B subjects are more likely to have a history of childhood aggression and conduct disorder and less likely to have a sustained response to treatment in comparison to Type A subjects ([21] Babor *et al.,* 1992). More recent studies also emphasize the role of externalizing disorders, such as conduct disorder and attention deficit hyperactivity disorder in increasing the risk for alcohol problems ([22] Kuperman *et al.,* 2001; [23] Bucholz *et al.,* 2017; [24] Groenman *et al.,* 2017). Cannabis and tobacco use are also associated with increased risk for concomitant alcohol problems ([23] Bucholz *et al*., 2017).

We studied a sample at risk for the development of alcohol use disorder on the basis of family history. Initial assessment was done on all subjects in the age range 12–21. These subjects have been followed over time with assessments every two years for up to 10 years. The present report evaluates the relationship of comorbid externalizing and internalizing disorders to age of onset of an AUD in a group of adolescents/young adults at high risk for AUDs. We also compare the onset of two alcohol milestones (age of first drink, age of first regular drinking) in groups divided by AUD severity. We hypothesized that persons developing AUDs following the onset of externalizing and internalizing disorders would show earlier onset than those without those baseline disorders. We also hypothesized that more severe AUDs would show an earlier onset of alcohol-related developmental milestones such as age of first drink and age of first regular drinking. The present report is one of the first we are aware of that tracks the development of AUDs in the context of multiple comorbid disorders in a high risk group, and it shows that some subjects are at great risk for alcohol problems in very early adolescence.

## METHODS

Our subjects were participants in the adolescent to young adult Prospective sample of COGA (*N* = 3286). The COGA study started in 1989 and families were recruited between 1989 and 1995. Each family was recruited through a proband with an alcohol use disorder (at that time [25] DSMIII-R and [26] Feighner), targeting successive admissions to treatment facilities. There was a family size requirement (at least two living first-degree relatives) with the idea of prioritizing larger families. All first-degree relatives were interviewed and families were extended through affected subjects (*i.e.,* the identification of an affected uncle of the proband would then lead to invitations for interview of that uncle’s family members). The subjects in the present study were offspring of the proband (or in some cases second-degree relatives). The response rate for recruitment was about 70% or more (with some inter-site variation). More information about the COGA study may be found in [23] Bucholz *et al.,* 2017 and [27] Reich *et al.,* 1998. All offspring in the age range (12–21) at the start of follow-up (2006–2007) were included. Offspring reaching the age of 12 during the course of the study (2006–2019) were also included. Subjects were interviewed at two-year intervals with the Semi-Structured Assessment for the Genetics of Alcoholism (SSAGA-IV) interview [28] Bucholz *et al*., 1994. The mean age at first interview was 16.1 (3.3 SD) and the mean age at last interview 23.1 (5.0). Subjects had an average of 4.0 interviews (1.7). 50.9% were female, 64.9% were EA and 30.9% AA. Ethnicity was assigned based on genotypic data, or by self-report if genotypes were not available. Subjects were members of a case family (proband with alcohol dependence −86.7% of subjects) or a comparison family (families recruited from medical or dental clinics or motor vehicle records with no selection for presence or absence of psychopathology). Non-drinkers were not excluded from the sample. The study was approved by The Indiana University Institutional Review Board (IRB) (project code: 1011004039R009, October 12, 2018). Written informed consent for the research was obtained from all participants in the study.

All subjects in the study were invited to participate in interviews at two-year intervals. Detailed information on participation is provided in [23] Bucholz *et al.,* 2017. Information on all available interviews for each subject was combined in the present analysis with age of onset assigned according to the earliest description of psychopathology and a judgment of severity based on the time when the most symptoms were described. Every subject with at least one complete interview was included in the analysis.

DSM-IV [29] diagnoses for all disorders were made algorithmically from SSAGA information. However for these analyses we also generated a DSM-5 [30] diagnosis for AUD in the following way. Individual alcohol symptoms were queried, starting with symptoms of DSM-IV alcohol dependence and alcohol abuse, adding craving and subtracting legal problems related to alcohol. Onset and offset of each symptom was recorded, making it possible to cluster symptoms that occurred by age. Thus the analyses presented here use DSM-5 AUD as an outcome variable while all other disorders are diagnosed by DSM-IV.

Diagnoses of externalizing and internalizing disorders at the baseline interview were also made algorithmically from the SSAGA using DSM-IV. Externalizing disorders included any of the following: ADHD, conduct disorder/antisocial personality disorder, oppositional defiant disorder, drug use disorder (including marijuana but not alcohol or tobacco). Internalizing disorders included major depression, panic disorder, obsessive-compulsive disorder, social phobia, and agoraphobia. Age of onset was determined for all comorbid disorders based on the SSAGA-IV.

Subjects were divided into groups based on whether they had an externalizing disorder or an internalizing disorder at the time of the baseline interview. The groups were: Externalizing, Internalizing, Both, or Neither. Alcohol use disorder diagnosis was then assessed at each interview period, using the DSM 5 distinctions for Mild AUD (2–3 symptoms), Moderate AUD (4–5 symptoms), and Severe AUD (6 or more symptoms. The age of onset was defined as the first age when the required number of symptoms occurred during the same year. If two interviews performed on the same subject at different times provided divergent estimates of age of onset, the earliest age was taken as correct. Subjects were stratified into mutually exclusive categories based on the most severe AUD diagnosis they received during any part of the follow-up period *(i.e.,* subjects with severe AUD were not counted in the Mild or Moderate AUD categories, though they may have aged through a time when they would have qualified for one or both of those diagnoses). Subjects with age of onset of AUD prior to age of onset of internalizing/externalizing disorders were excluded from analysis.

We also performed a sensitivity analysis (for model dependence) in which all subjects with externalizing (with or without internalizing) were compared with all subjects without externalizing; likewise subjects with internalizing (with or without externalizing) were compared with all subjects without internalizing (see Supplementary materials). An externalizing-internalizing interaction term was included in this analysis.

### Statistical Methods

A Cox proportional hazards model was used to test the relationship of baseline externalizing or internalizing diagnoses to later onset of an AUD. All Cox model analyses were adjusted for sex, ethnicity, family membership, and case/comparison status. Survival curves were estimated by Kaplan-Meier plots. Ages of onset for alcohol milestones were compared using ANOVA and t-test.

## RESULTS

Overall, 43.0% of the sample met criteria for a diagnosis of either Mild, Moderate, or Severe AUD by the end of the observation period (1416/3286; Table 1).

**Figure 1.**
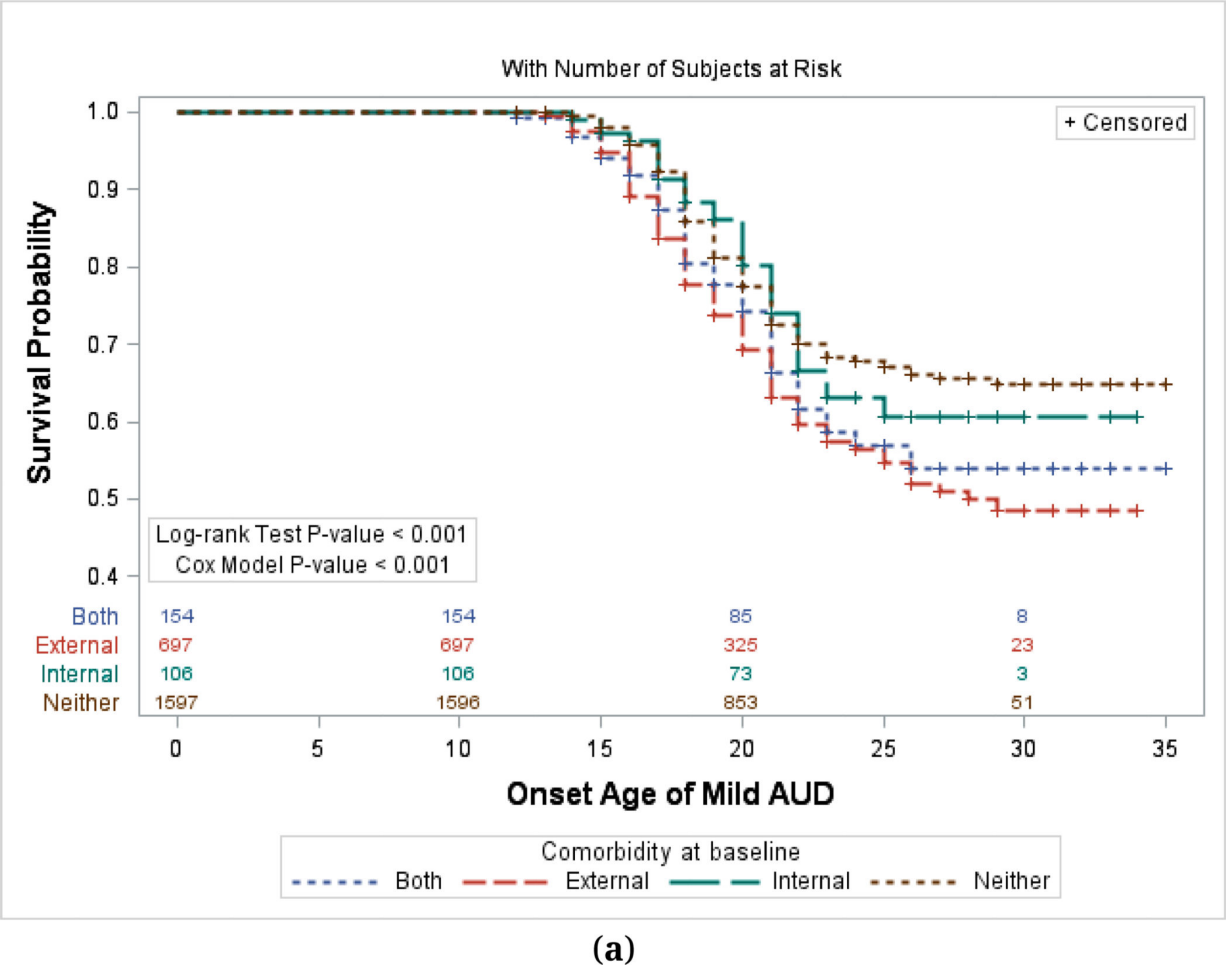

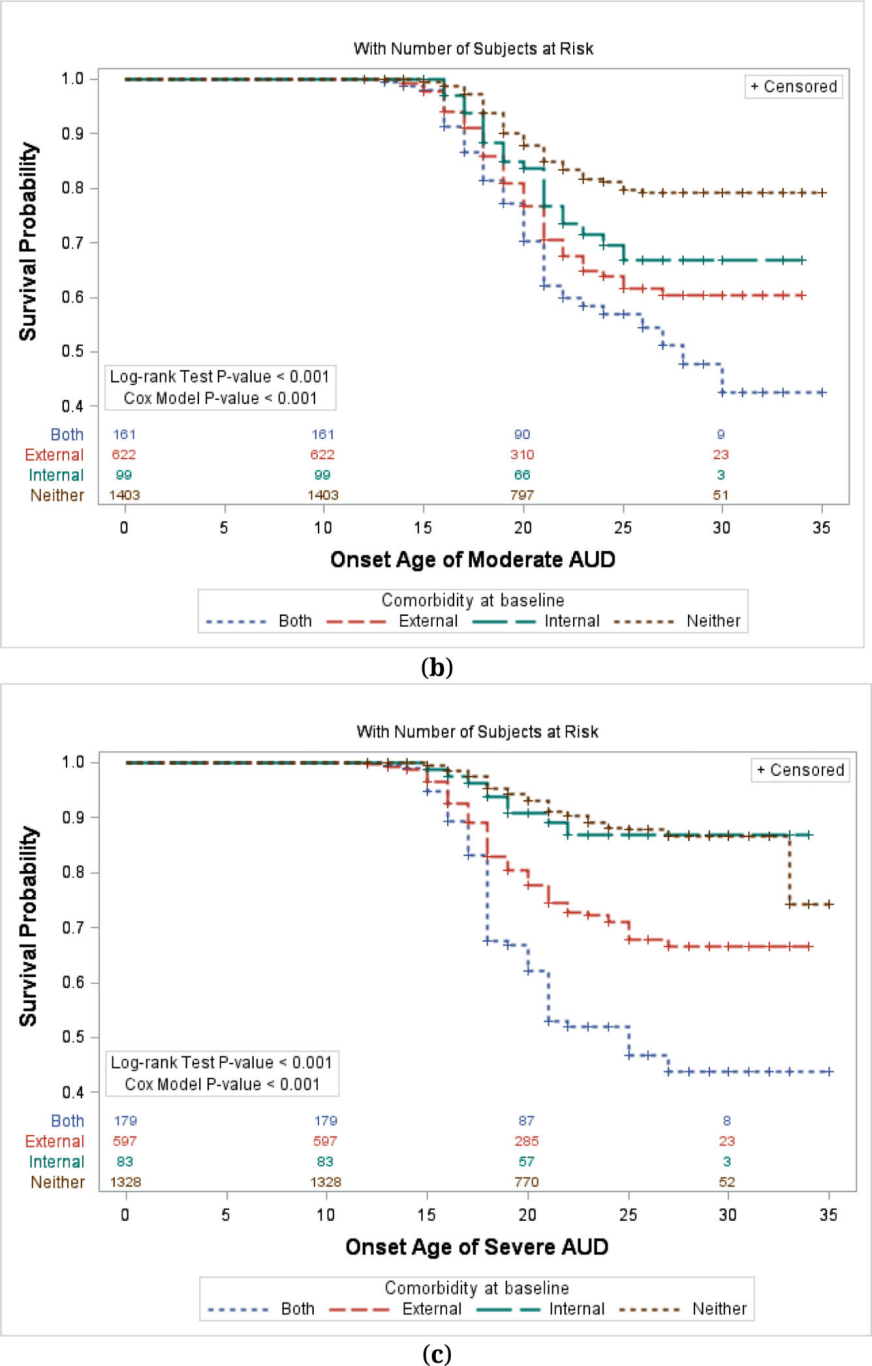
**(a)** Kaplan-Meier Plot by Prior Comorbidity—Mild AUD. (**b**) Kaplan-Meier Plot by Prior Comorbidity—Moderate AUD. (**c**) Kaplan-Meier Plot by Prior Comorbidity—Severe AUD. Numbers in the Figure represent the N of subjects at risk at various ages.

At the time of the baseline interview, 982/3286 subjects had an externalizing diagnosis (29.9%); 140/3286 subjects had an internalizing diagnosis (4.3%), 286 had both (8.7%) and 1878 had neither (57.2%) (Table 1). All covariates (sex, ethnicity, family type) had significant relationships to age of onset in subjects with either mild, moderate, or severe AUD (Table 2). The association of any comorbid disorder and presence of Alcohol Use Disorder was significant overall (chi-square *p*-value < 0.0001), and there was a significant effect of comorbidity on age of onset as well (*p* < 0.001 for each type of AUD, Figure 1a–c). Among subjects with an externalizing disorder only at baseline, 515/982 (52.4%) had some type of AUD during the follow-up period. Among subjects with an internalizing disorder only at baseline 66/140 (47.1%) had an AUD. Among subjects with both externalizing and internalizing, 182/286(63.6%) had an AUD. In comparison, subjects with neither type of disorder had an AUD rate of 34.7% (653/1878).

Figure 1 shows onset of alcohol use disorders (AUDs) in subjects stratified by initial diagnoses of Externalizing disorder, Internalizing disorder, Both, or Neither. Figures 1a–c show onset of mild, moderate, and severe AUDs respectively. For each type of AUD, the relationship with comorbid disorders is significant by Log-rank test (*p* < 0.001) and Cox Proportional Hazards (*p* < 0.001).

Age of onset comparisons are shown in Kaplan-Meier Plots (Figure 1a–c). Each of these shows significant effects of comorbidity by Log-rank Test (*p* < 0.001 for each). The plots do not include a covariate effect but we have also achieved similar results by the Cox model adjusting for covariate effects (*p* < 0.001 for each; Table 2). The statistical effect of comorbidity is generally greatest in the development of Severe AUD and least in Mild AUD based on the hazard ratios in the different comorbidity types (Table 2). The three groups are significantly different from each other in the strength of the comorbidity effect (Severe *vs* Moderate, *p* < 0.001; Severe *vs* Mild, *p* < 0.001; Moderate *vs* Mild, *p* < 0.001).

The sensitivity analysis (Supplementary Table S1) showed a clear effect of externalizing on age of onset in mild AUD, moderate AUD, and severe AUD (*p* < 0.001 for each). For internalizing, there was an effect in moderate AUD (*p* < 0.011) and severe AUD (*p* < 0.001). No statistical interaction was seen between the effect of externalizing and the effect of internalizing.

**Figure 2.**
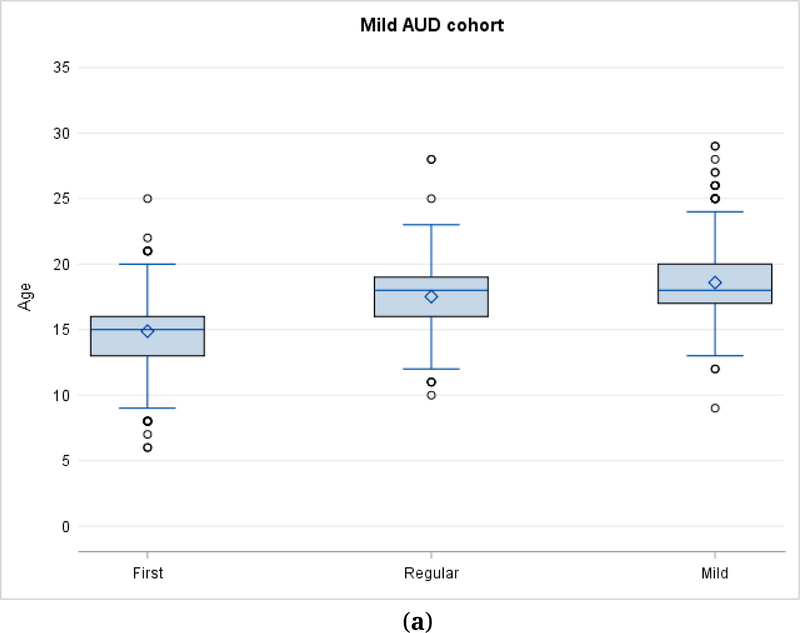

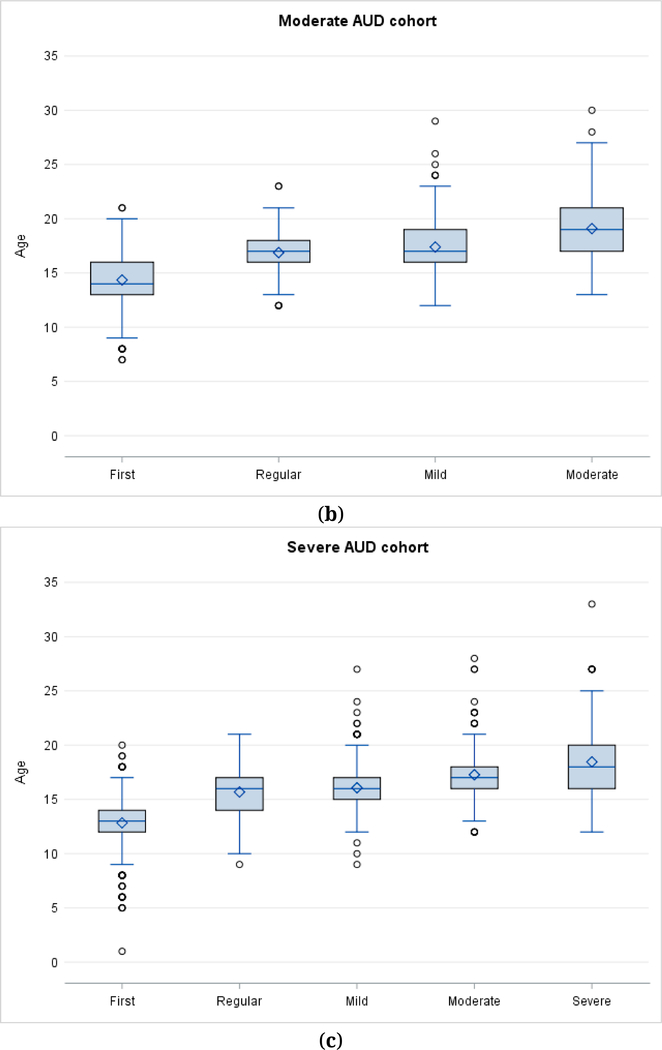
**(a)**Age of onset for drinking milestones in Mild AUD. (**b**) Age of onset for drinking milestones in Moderate AUD. (**c**) Age of onset of drinking milestones in Severe AUD.

Age of onset distributions are presented for Mild AUD (Figure 2a), Moderate AUD (Figure 2b), and Severe AUD (Figure 2c). The distributions include drinking milestones (first drink, first regular drinking) as well as onset ages for the diagnoses of Mild AUD (Figure 2a–c), Moderate AUD (Figure 2b,c) and Severe AUD (Figure 2c only). As noted above, the study samples are independent of each other for analytic purposes, and are classified according to the most severe disorder that the subject met criteria for during the observation period.

Figure 2 shows drinking milestones in subjects who developed an alcohol use disorder.

Figure 2a–c show mean, median, interquartile range, and outliers for subjects with mild (*N* = 684), moderate (*N* = 415) and severe (*N* = 317) alcohol use disorder. Subjects are classified in a cohort according to the most severe form of disorder they manifested during the observation period. In Figure 2b milestones for the moderate group include the age when they would have been first classified as showing a mild AUD. In Figure 2c milestones for the severe group include the ages when they would have been first classified as showing a mild or moderate AUD.

**Figure 3.**
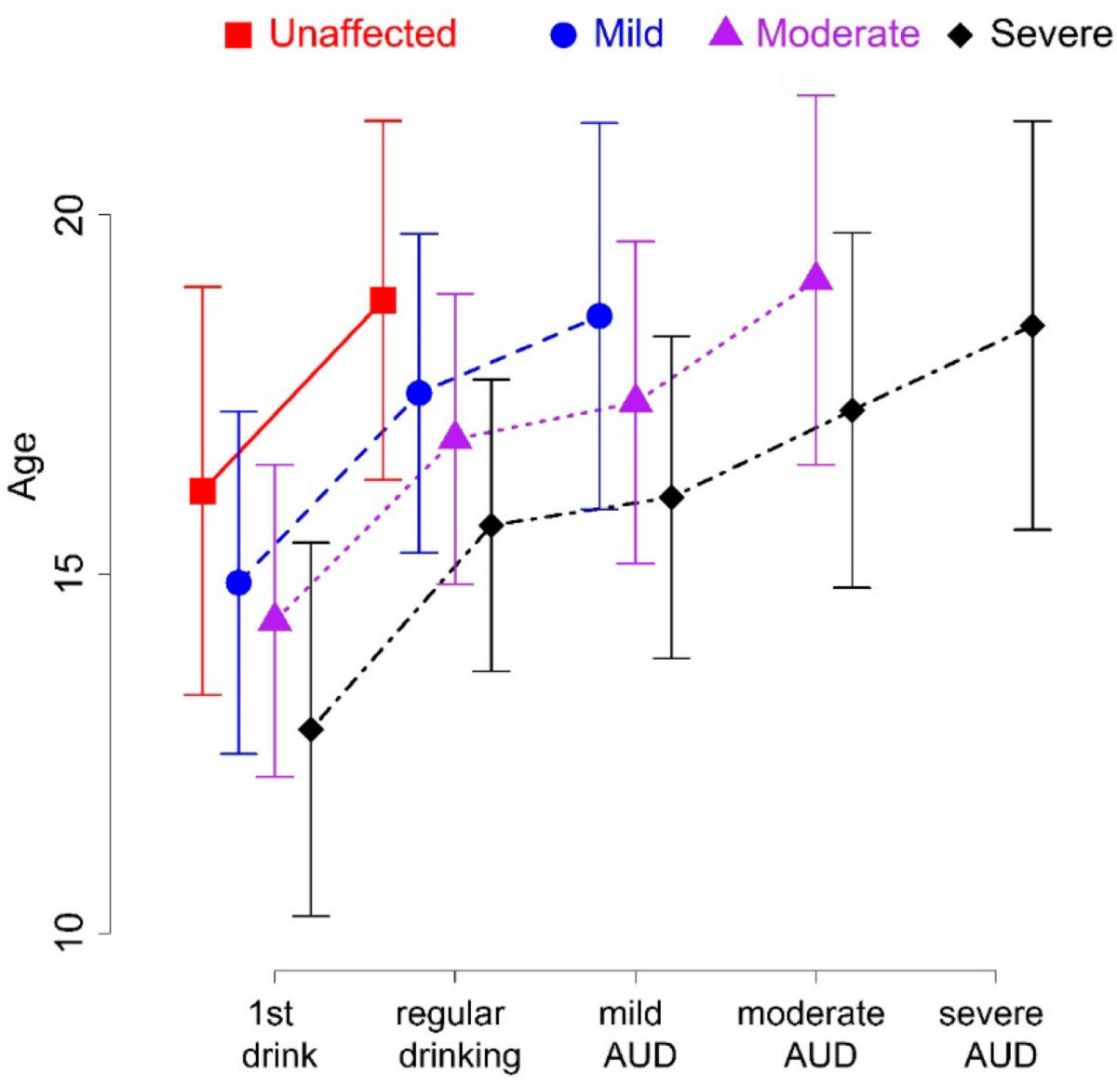
Comparison of drinking milestones in four groups divided by severity of alcohol problems.

We used ANOVA and i-test to detect the correlation between the onset of drinking milestones in the four diagnostic groups. The mean age of first drink progresses from 16.2 in Unaffected to 14.9 in Mild to 14.4 in Moderate to 12.8 in Severe (*p* < 0.001). The mean age of first regular drinking progresses from 18.8 in Unaffected to 17.5 in Mild to 16.9 in Moderate to 15.7 in Severe (*p* < 0.001). The mean age for meeting criteria for Mild AUD progresses from 18.6 in Mild to 17.4 in Moderate to 16.1 in Severe (*p* < 0.001). The mean age for meeting criteria for Moderate AUD progresses from 19.1 in Moderate to 17.3 in Severe (*p* < 0.001). The age of onset for Severe AUD is 18.5. This age relationship is detailed in Figure 3.

Figure 3 represents the onset of alcohol use and alcohol problems in 3286 adolescents observed over a ten year period. It includes 1870 who remained unaffected, 684 who developed mild alcohol use disorder, 415 who developed moderate alcohol use disorder, and 317 who developed severe alcohol use disorder.

The ANOVA for onset of first drink among the unaffected, mild, moderate, and severe cohorts shows *p* < 0.001. The ANOVA for onset of regular drinking among the unaffected, mild, moderate, and severe cohorts shows *p* < 0.001. The ANOVA for onset age of mild AUD among the mild, moderate, and severe cohorts shows *p* < 0.001. The *t*-test for onset age of moderate AUD between the moderate and severe cohorts shows *p* < 0.001.

## DISCUSSION

These data suggest a strong effect of externalizing and internalizing disorders on prevalence and age of onset of Alcohol Use Disorder among adolescents/young adults at risk for the development of AUD on the basis of family history. Externalizing disorders were clearly associated with an increased risk for AUD and for earlier development of AUD. Internalizing disorders by themselves did not show a significant effect, but in combination with externalizing disorders they were associated with an earlier onset for severe AUD (in comparison to externalizing disorders alone). When we considered all internalizing disorders together (with or without externalizing disorders) a clear effect on onset of moderate AUD was seen as well. By the end of the follow-up period, more than 60% of young people with both externalizing and internalizing disorders at baseline had developed alcohol dependence in comparison with about 30% of young people with neither type of comorbid disorder. The effect of comorbidity was stronger in more severe forms of AUD, with a 6-fold increase in risk for Severe AUD among subjects with both externalizing and internalizing disorders compared to subjects with neither form of comorbid disorder.

There was also evidence for an earlier developmental course in more severe forms of AUD compared to less severe. Persons with Severe AUD were likely to have their first full drink prior to the age of 13 and be drinking regularly prior to age 16 and experiencing 1–2 alcohol problems by that same age. In contrast young people who did not demonstrate any AUD were likely to have their first drink at 16 and start regular drinking just prior to age 19. Median and mean ages of onset for each type of AUD were 18–19, though the range extended through the follow-up period.

Those at greatest risk for an AUD were males of European descent from an alcohol-dependent proband family with one or more childhood onset psychiatric diagnoses. Those at least risk were females of African-American ancestry from a non-case family with no childhood onset diagnosis.

Limitations of the study include the fact that all analyses are based on self-report and there is no independent corroboration of diagnoses or symptoms. Subjects interviewed in their late 20s may have had more difficulty with accurate reporting of events in early teenage years in comparison to subjects in their mid-teens. Retention rate from baseline interview to two-year interview was 85%, the majority of subjects completed at least four interviews ([23] Bucholz *et al.,* 2017). Families in the COGA study tend to be densely affected and results may not be generalizable to persons with alcohol use disorder in the general population. The subjects were ascertained at 7 University-based clinical sites (State University of New York, Brooklyn, University of Iowa, Iowa City, Indiana University School of Medicine, Indianapolis, Washington University in St. Louis, University of California San Diego, University of Connecticut, Hartford, and Howard University, Washington DC) and the populations studied reflect those sites.

The magnitude of these effects was substantial, and this information may be helpful in targeting efforts at education and prevention. In this sample most of the AUD-affected subjects had a comorbid psychiatric disorder at baseline. Many such subjects may come to clinical attention for their childhood-onset disorders and it may be worth educational efforts targeting AUD, especially for those at increased familial risk. It has been argued, though, that more intensive interventions are not likely to be cost- effective at this time ([13] Glantz *et al.,* 2009). It seems to be of value to continue to try to quantify risk in various clinically and biologically identifiable groups. Polygenic risk scores, especially as they increase in power with data from expanding clinical samples, will likely be of use ([31] Fullerton and Nurnberger, 2019). It would also be of value to attempt to separate AUD effects from other forms of SUD, since we know that they are highly comorbid in many samples, including the sample studied here.

## Supplementary Material

supplemental File 1

## Figures and Tables

**Table 1.** Baseline characteristics (*N* = 3286).

Subject Variable	Mean (SD)
Age at first interview	16.13 (3.29)
Age at last interview	23.13 (4.97)
Number of interviews	4.02 (1.73)
Gender	
Female	1673 (50.91%)
Male	1613 (49.09%)
Ethnicity	
EA	2133 (64.91%)
AA	1016 (30.92%)
Other	155 (4.21%)
Family type	
Case	2848 (86.67%)
Control	438 (13.33%)
Any type of AUD	
Yes	1416 (43.09%)
No	1870 (56.91%)
Comorbidity	
Externalizing only	982 (29.88%)
Internalizing only	140 (4.26%)
Both	286 (8.70%)
Neither	1878 (57.15%)

**Table 2.** Hazard ratio and 95% CI-Cox model.

Comorbidity	Outcome	Description	HR (95% CI)
Comorbidity at baseline	Onset age of mild AUD	Gender: Female *vs* Male	0.71 (0.61, 0.83)
		Ethnicity: African American *vs* European American	0.65 (0.55, 0.77)
		Ethnicity: African American *vs* Other	1.11 (0.68, 1.79)
		Ethnicity: European American *vs* Other	1.69 (1.06, 2.71)
		Family type: Control *vs* Case	0.74 (0.59, 0.93)
		Comorbidity at baseline: Both *vs* External	0.89 (0.65, 1.21)
		Comorbidity at baseline: Both *vs* Internal	1.35 (0.86, 2.10)
		Comorbidity at baseline: Both vs Neither	1.39 (1.04, 1.87)
		Comorbidity at baseline: External *vs* Internal	1.51 (1.04, 2.20)
		Comorbidity at baseline: External *vs* Neither	1.57 (1.33, 1.85)
		Comorbidity at baseline: Internal *vs* Neither	1.04 (0.72, 1.49)
	Onset age of moderate AUD	Gender: Female *vs* Male	0.61 (0.50, 0.75)
		Ethnicity: African American *vs* European American	0.45 (0.35, 0.57)
		Ethnicity: African American *vs* Other	0.43 (0.27, 0.70)
		Ethnicity: European American vs Other	0.97 (0.62, 1.51)
		Family type: Control *vs* Case	0.38 (0.25, 0.56)
		Comorbidity at baseline: Both *vs* External	1.32 (0.97, 1.79)
		Comorbidity at baseline: Both *vs* Internal	1.84 (1.15, 2.95)
		Comorbidity at baseline: Both *vs* Neither	2.80 (2.07, 3.77)
		Comorbidity at baseline: External *vs* Internal	1.39 (0.91, 2.14)
		Comorbidity at baseline: External *vs* Neither	2.12 (1.70, 2.64)
		Comorbidity at baseline: Internal *vs* Neither	1.52 (1.00, 2.31)
Comorbidity at baseline	Onset age of severe AUD	Gender: Female *vs* Male	0.70 (0.56, 0.88)
		Ethnicity: African American *vs* European American	0.37 (0.28, 0.49)
		Ethnicity: African American *vs* Other	0.23 (0.14, 0.36)
		Ethnicity: European American *vs* Other	0.62 (0.41, 0.94)
		Family type: Control *vs* Case	0.32 (0.19, 0.52)
		Comorbidity at baseline: Both *vs* External	1.88 (1.41, 2.51)
		Comorbidity at baseline: Both *vs* Internal	6.01 (3.00, 12.04)
		Comorbidity at baseline: Both *vs* Neither	6.08 (4.51, 8.21)
		Comorbidity at baseline: External *vs* Internal	3.20 (1.62, 6.32)
		Comorbidity at baseline: External *vs* Neither	3.24 (2.49, 4.21)
		Comorbidity at baseline: Internal *vs* Neither	1.01 (0.51, 2.00)
