# Supplementary material for: Development of Alcohol Use Disorder as a Function of Age, Severity, and Comorbidity with Externalizing and Internalizing Disorders in a Young Adult Cohort
Source: J Psychiatr Brain Sci. Author manuscript; Available in PMC 2019 Dec 18. (PMC6919651; doi:10.20900/jpbs.20190016)
Supplement: supplemental File 1 [file NIHMS1056454-supplement-supplemental_File_1.pdf]

Supplementary Materials

Sensitivity Analysis

We recoded the baseline comorbidity as follows:

```
if comor_bsl in ("External", "Both") then EXT="Yes"; else EXT="No";
if comor_bsl in ("Internal", "Both") then INT="Yes"; else INT="No";
```

Then we test whether the interaction of EXT and INT is significant. If it is not, we remove it from the model.

Table S1. Mild AUD.

Analysis of Maximum Likelihood Estimates								
**Parameter**		**DF**	**Parameter Estimate**	**Standard Error**	**Chi-Square**	**Pr > ChiSq**	**Hazard Ratio**	**Label**
**dm1c**	**Female**	1	−0.33699	0.07746	18.9275	<0.0001	0.714	dm1c Female
**ethnicity**	**African American**	1	0.09709	0.24669	0.1549	0.6939	1.102	ethnicity African American
**ethnicity**	**European American**	1	0.52354	0.24029	4.7470	0.0294	1.688	ethnicity European American
**TypeFAM**	**0**	1	−0.30609	0.11626	6.9321	0.0085	0.736	TypeFAM 0
**EXT**	**No**	1	−0.43215	0.08060	28.7493	<0.0001	0.649	EXT No
**INT**	**No**	1	0.05629	0.12035	0.2188	0.6400	1.058	INT No

Hazard Ratios for dm1c			
**Description**	**Point Estimate**	**95% Wald Confidence Limits**	
**dm1c Female vs Male**	0.714	0.613	0.831

Hazard Ratios for ethnicity			
**Description**	**Point Estimate**	**95% Wald Confidence Limits**	
**ethnicity African American *vs* European American**	0.653	0.551	0.773
**ethnicity African American *vs* Other**	1.102	0.679	1.787
**ethnicity European American *vs* Other**	1.688	1.054	2.703

Hazard Ratios for TypeFAM			
**Description**	**Point Estimate**	**95% Wald Confidence Limits**	
**TypeFAM 0 *vs* 1**	0.736	0.586	0.925

**Hazard Ratios for EXT**			
**Description**	**Point Estimate**	**95% Wald Confidence Limits**	
**EXT No vs Yes**	0.649	0.554	0.760

**Hazard Ratios for INT**			
**Description**	**Point Estimate**	**95% Wald Confidence Limits**	
**INT No *vs* Yes**	1.058	0.836	1.339

Table S2. Moderate AUD.

**Analysis of Maximum Likelihood Estimates**								
**Parameter**		**DF**	**Parameter Estimate**	**Standard Error**	**Chi-Square**	**Pr > ChiSq**	**Hazard Ratio**	**Label**
**dm1c**	**Female**	1	−0.49120	0.10135	23.4876	<0.0001	0.612	dm1c Female
**ethnicity**	**African American**	1	−0.84174	0.24302	11.9970	0.0005	0.431	ethnicity African American
**ethnicity**	**European American**	1	−0.03497	0.22664	0.0238	0.8774	0.966	ethnicity European American
**TypeFAM**	**0**	1	−0.97938	0.20410	23.0268	<0.0001	0.376	TypeFAM 0
**EXT**	**No**	1	−0.72638	0.10289	49.8354	<0.0001	0.484	EXT No
**INT**	**No**	1	−0.32432	0.12724	6.4967	0.0108	0.723	INT No

**Hazard Ratios for dm1c**			
**Description**	**Point Estimate**	**95% Wald Confidence Limits**	
**dm1c Female *vs* Male**	0.612	0.502	0.746

**Hazard Ratios for ethnicity**			
**Description**	**Point Estimate**	**95% Wald Confidence Limits**	
**ethnicity African American *vs* European American**	0.446	0.353	0.565
**ethnicity African American *vs* Other**	0.431	0.268	0.694
**ethnicity European American *vs* Other**	0.966	0.619	1.506

Hazard Ratios for TypeFAM			
**Description**	**Point Estimate**	**95% Wald Confidence Limits**	
**TypeFAM 0 *vs* 1**	0.376	0.252	0.560

Hazard Ratios for EXT			
**Description**	**Point Estimate**	**95% Wald Confidence Limits**	
**EXT No *vs* Yes**	0.484	0.395	0.592

Hazard Ratios for INT			
**Description**	**Point Estimate**	**95% Wald Confidence Limits**	
**INT No *vs* Yes**	0.723	0.563	0.928

Table S3. Severe AUD.

Analysis of Maximum Likelihood Estimates								
**Parameter**		**DF**	**Parameter Estimate**	**Standard Error**	**Chi-Square**	**Pr > ChiSq**	**Hazard Ratio**	**Label**
**dm1c**	**Female**	1	−0.35835	0.11508	9.6962	0.0018	0.699	dm1c Female
**ethnicity**	**African American**	1	−1.46388	0.23796	37.8449	<0.0001	0.231	ethnicity African American
**ethnicity**	**European American**	1	−0.46109	0.21186	4.7367	0.0295	0.631	ethnicity European American
**TypeFAM**	**0**	1	−1.13628	0.25066	20.5498	<0.0001	0.321	TypeFAM 0
**EXT**	**No**	1	−1.26470	0.12310	105.5528	<0.0001	0.282	EXT No
**INT**	**No**	1	−0.51987	0.13301	15.2770	<0.0001	0.595	INT No

Hazard Ratios for dm1c			
**Description**	**Point Estimate**	**95% Wald Confidence Limits**	
**dm1c Female vs Male**	0.699	0.558	0.876

Hazard Ratios for ethnicity			
**Description**	**Point Estimate**	**95% Wald Confidence Limits**	
**ethnicity African American *vs* European American**	0.367	0.277	0.487
**ethnicity African American *vs* Other**	0.231	0.145	0.369
**ethnicity European American *vs* Other**	0.631	0.416	0.955

Hazard Ratios for TypeFAM			
**Description**	**Point Estimate**	**95% Wald Confidence Limits**	
**TypeFAM 0 *vs* 1**	0.321	0.196	0.525

Hazard Ratios for EXT			
**Description**	**Point Estimate**	**95% Wald Confidence Limits**	
**EXT No *vs* Yes**	0.282	0.222	0.359

Hazard Ratios for INT			
**Description**	**Point Estimate**	**95% Wald Confidence Limits**	
**INT No *vs* Yes**	0.595	0.458	0.772
